# Microwave background temperature at a redshift of 6.34 from H_2_O absorption

**DOI:** 10.1038/s41586-021-04294-5

**Published:** 2022-02-02

**Authors:** Dominik A. Riechers, Axel Weiss, Fabian Walter, Christopher L. Carilli, Pierre Cox, Roberto Decarli, Roberto Neri

**Affiliations:** 1 I. Physikalisches Institut, Universität zu Köln, Köln, Germany; 2 Max-Planck-Institut für Radioastronomie, Bonn, Germany; 3 Max-Planck-Institut für Astronomie, Heidelberg, Germany; 4 National Radio Astronomy Observatory, Pete V. Domenici Array Science Center, Socorro, NM USA; 5 Sorbonne Université, UPMC Université Paris 6 and CNRS, UMR 7095, Institut d’Astrophysique de Paris, Paris, France; 6 INAF - Osservatorio di Astrofisica e Scienza dello Spazio, Bologna, Italy; 7 Institut de Radioastronomie Millimétrique, Saint Martin d’Héres, France

**Keywords:** Cosmology, Early universe

## Abstract

Distortions of the observed cosmic microwave background provide a direct measurement of the microwave background temperature at redshifts from 0 to 1 (refs. ^1,2^). Some additional background temperature estimates exist at redshifts from 1.8 to 3.3 based on molecular and atomic line-excitation temperatures in quasar absorption-line systems, but are model dependent^3^. No deviations from the expected (1 + *z*) scaling behaviour of the microwave background temperature have been seen^4^, but the measurements have not extended deeply into the matter-dominated era of the Universe at redshifts *z* > 3.3. Here we report observations of submillimetre line absorption from the water molecule against the cosmic microwave background at *z* = 6.34 in a massive starburst galaxy, corresponding to a lookback time of 12.8 billion years (ref. ^5^). Radiative pumping of the upper level of the ground-state ortho-H_2_O(1_10_–1_01_) line due to starburst activity in the dusty galaxy HFLS3 results in a cooling to below the redshifted microwave background temperature, after the transition is initially excited by the microwave background. This implies a microwave background temperature of 16.4–30.2 K (1*σ* range) at *z* = 6.34, which is consistent with a background temperature increase with redshift as expected from the standard ΛCDM cosmology^4^.

## Abstract (web summary)

Measurement of the cosmic microwave background temperature using H_2_O absorption at a redshift of 6.34 is reported, the results of which were consistent with those from standard ΛCDM cosmology.

## Main

**Fig. 1:**
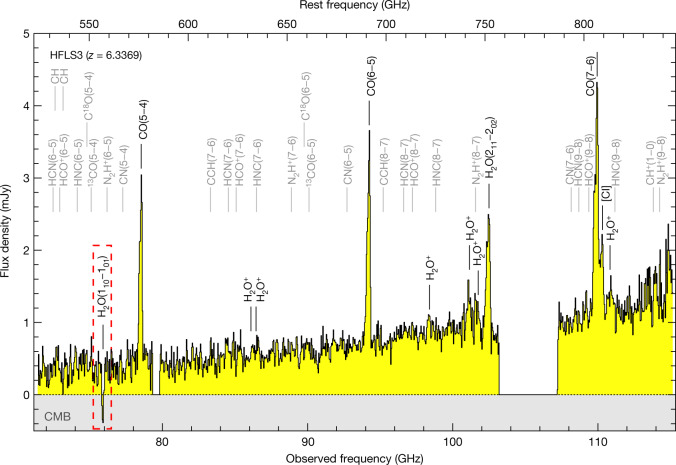
Broad-band, 3-mm spectroscopy of the starburst galaxy HFLS3 at a redshift of 6.34 with NOEMA. Black/yellow histogram, NOEMA spectroscopy data, binned to 40-MHz (158 km s^−1^ at 75.9 GHz) spectral resolution. Expected frequencies of molecular and atomic lines at the redshift of HFLS3 are indicated, with the dominant species labelled in black. The dashed red box indicates the frequency range of the ortho-H_2_O(1_10_−1_01_) line, which is detected in absorption against the CMB.

**Fig. 2:**
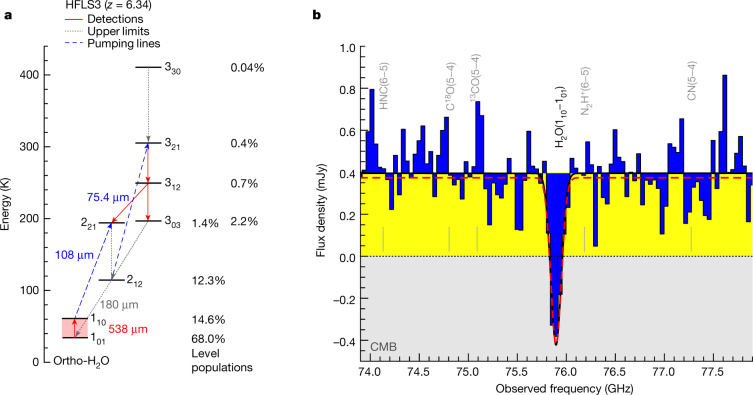
H_2_O line and continuum properties and modelling of HFLS3. Ortho-H_2_O energy-level structure (**a**, red solid arrows are detected transitions, grey dotted lines are upper limits and blue dashed arrows are pumping transitions, with observed and model-predicted absorption/emission lines indicated as upward/downward arrows, respectively; percentages are the level populations in the model) and zoom-in on the H_2_O line at the same spectral resolution as in Fig. 1 to show that the line absorbs into the CMB (**b**, blue shading added for emphasis). The black curve is a fit to the spectrum. The red dashed curve is the best-fit radiative transfer model. Source data

**Extended Data Fig. 1:**
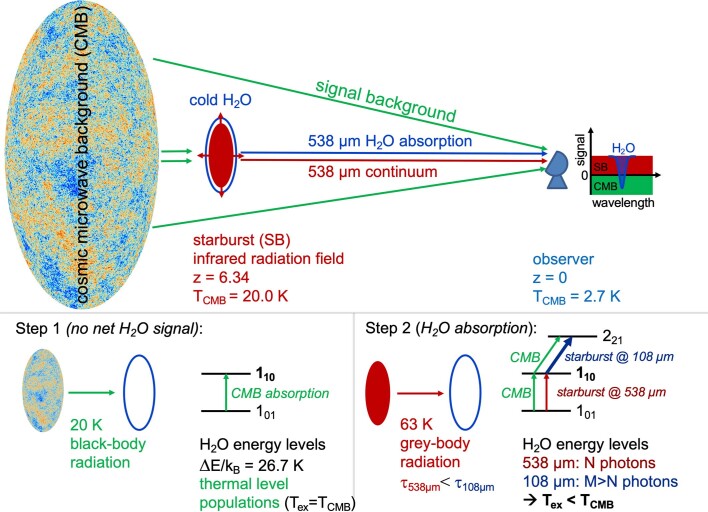
Combined effect of CMB absorption and starburst radiation field on the strength of the H_2_O(1_10_–1_01_) line in HFLS3. Top, the cold H_2_O vapour is exposed to the CMB radiation field^4^, which has the shape of a black-body function (*T*_CMB_ = 20.0 K at *z* = 6.34), and the starburst infrared radiation field, which has the shape of a grey-body function (*T*_dust_ = 63 K). NOEMA observed the signal in contrast to the CMB and therefore detects only the dust emission from the starburst and the H_2_O line, but not the CMB itself (which therefore fills the region below zero flux density, as seen by the telescope). Bottom left, as the energy-level difference for the H_2_O(1_10_–1_01_) line is only 26.7 K, there are sufficient CMB photons at *z* = 6.34 to thermalize the level population between both levels, such that *T*_ex_ is the same as *T*_CMB_ in equilibrium. Therefore no H_2_O emission or absorption will be observed, despite the presence of a ‘seed’ population in the upper level. Bottom right, the radiation field of the starburst alters the level populations towards increased higher-level populations. Owing to the grey-body shape of its spectral energy distribution, more photons are available at 108 μm to increase the 2_21_ level population from the 1_10_ state than there are 538-μm photons available to increase the 1_10_ level population from the 1_01_ state, relative to the ‘seed’ population provided by the absorption of CMB photons. Therefore the relative population of the 1_10_ and 1_01_ levels is lower than in thermal equilibrium, such that the resulting *T*_ex_ is lower than *T*_CMB_. As a result, the H_2_O(1_10_–1_01_) line is observed in absorption towards the CMB due to the negative temperature contrast—as observed towards HFLS3.

We used the Northern Extended Millimeter Array (NOEMA) to obtain a sensitive scan across the 3-mm atmospheric window towards the *z* = 6.34 massive dusty starburst galaxy HFLS3 (also known as 1HERMES S350 J170647.8+584623; see Methods)^5^. These observations reveal a broad range of emission features dominated by the CO, H_2_O and H_2_O^+^ molecules and atomic carbon, on top of thermal dust continuum emission that is rising with frequency consistent with a dust temperature of *T*_dust_ = ${63.3}_{-5.8}^{+5.4}$ K (refs. ^5,6^) (Fig. 1). The spectrum also shows a deep absorption feature due to the ortho-H_2_O(1_10_–1_01_) ground-state transition at rest-frame 538 μm (observed at 3.95 mm, or 75.9 GHz). This absorption is about two times stronger than the continuum emission from the starburst at the same frequency (Fig. 2). For this effect to occur, a substantial population of the ortho-H_2_O 1_10_ level (which lies 26.7 K above the 1_01_ ground state) has to be excited by cosmic microwave background (CMB) photons as a basis for pumping of this level by the starburst infrared radiation field (see Extended Data Fig. 1). The effect becomes observable towards HFLS3 because of the warm CMB at this redshift, which is predicted to be *T*_CMB_ = 20.0 K at *z* = 6.34 based on the standard ΛCDM cosmology (where *T*_CMB_(*z*) = *T*_CMB_(*z* = 0)*(1 + *z*)^(1−*β*)^, *T*_CMB_(*z* = 0) = 2.72548 ± 0.00057 K (ref. ^7^) and the power-law index *β* = 0). The absorption of photons from the CMB radiation field appreciably populates the H_2_O 1_10_ level. The intense infrared radiation field from the starburst then preferentially de-populates the 1_10_ level through radiative pumping, resulting in a deficit in the 1_10_ level compared with 1_01_ relative to a thermal distribution. In combination, these two processes result in an excitation temperature *T*_ex_ of the H_2_O(1_10_–1_01_) line that is lower than *T*_CMB_, such that the line becomes observable in absorption against the CMB. As the effect depends on the strength of the CMB radiation field, it can be used to measure *T*_CMB_ for galaxies that have well-measured dust spectral energy distributions and dust continuum sizes, as is the case for HFLS3.

**Fig. 3:**
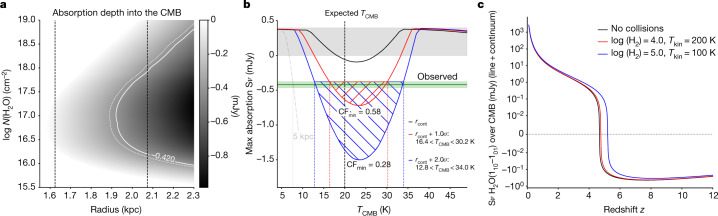
Radiative transfer models for HFLS3 and constraints on the CMB temperature. **a**, Model grid for the predicted line-absorption strength for *T*_CMB_(*z* = 6.34) = 20.0 K (greyscale) as a function of H_2_O column density (*y* axis) and radius of the dust-emission region at 108 μm (*x* axis). The white curves show the parameter space allowed by the measurement (solid line) and the −1*σ* r.m.s. uncertainty region (dotted line). The dashed black lines show the measured continuum size (left) and +1*σ* r.m.s. uncertainty region (right). The overlapping region between the white boundary (that is, the minimum allowed absorption strength) and the size measurement (that is, the minimum required emitting area at 100% covering fraction) is the allowed parameter space for the absorption strength within 1*σ* r.m.s. The minimum required radius at *N*(H_2_O)  ~ 10^17^ cm^−2^ is due to a minimum in *T*_ex_ in the models. **b**, Constraints on *T*_CMB_ for the observed absorption strength (green line and shaded region) at the minimum size compatible with the observations (**a**), based on the same models (red/blue shaded regions are the allowed ranges within the source radius +1*σ*/+2*σ* r.m.s.). The source radius at face value (black line), as well as the −1*σ* and −2*σ* r.m.s. regions (not shown), are ruled out by the observations. The minimum filling factor of the dust emission region CF_min_ is indicated for the +1*σ* and +2*σ* r.m.s. regions. The grey dashed line shows a model assuming a continuum radius of 5 kpc, which provides a conservative lower limit on *T*_CMB_. **c**, Observability of the H_2_O absorption as a function of redshift for three solutions allowed by the data without and with collisional excitation. The effect becomes observable at *z* ~ 4.5 and remains visible at similar strength to *z* > 12. The lower-redshift limit is higher in cases where collisional excitation is important, but the impact is minor below *n*(H_2_) = 10^5^ cm^−3^.

To understand the effect, we have calculated a series of spherically symmetric RADEX^8^ models over a wide range of H_2_O column densities, assuming purely radiative excitation (Figs. 2 and 3; see Methods for additional details). Exposing a cold, H_2_O-bearing region associated with HFLS3 to the black-body CMB radiation field at *T*_CMB_(*z* = 6.34), the models suggest that 77.2% of the molecules will be in the 1_01_ ground state and 20.3% will be in the upper 1_10_ state, and all H_2_O transitions have an excitation temperature *T*_ex_ equal to *T*_CMB_. As a result of this zero temperature contrast, no H_2_O emission or absorption would be observable, despite the fact that the H_2_O rotational ladder is excited by the CMB radiation. However, this picture changes when the same region is also exposed to the infrared radiation field emitted by the starburst nucleus of HFLS3, as the latter does not follow a single-black-body radiation pattern. Indeed, the infrared spectral energy distribution of HFLS3 reaches its peak intensity at ${73.3}_{-1.3}^{+1.6}$ μm and can be approximated by a grey body with a Rayleigh–Jeans slope of *β*_IR_ = ${1.94}_{-0.09}^{+0.07}$. This is due to the presence of dust at multiple temperatures and an increasing dust optical depth towards shorter wavelengths^5,6^. In this case, the level populations of the 1_01_ and 1_10_ states will deviate from the single-temperature thermal equilibrium population and change to 68.0% and 14.6%, respectively, for the solution shown in Fig. 2, resulting in an excitation temperature *T*_ex_ of only 17.4 K for this transition. Owing to the Δ*J* = 1 selection rule for photon emission/absorption, only three ortho-H_2_O transitions contribute to the modification of populations in the 1_01_ and 1_10_ levels, namely, the 538-μm 1_10_–1_01_ and 180-μm 2_12_–1_01_ transitions affecting the former, and the 108-μm 2_21_–1_10_ transition affecting the latter (see Fig. 2; the 2_21_–1_01_ transition is forbidden). The over-proportional de-population of the 1_10_ level occurs because the H_2_O(2_21_–1_10_) transition at 108 μm dominates the modification of the level population^9,10^. This transition lies near the peak of the dust spectral energy distribution, where the dust emission has a higher optical depth than at 538 μm, where the H_2_O(1_10_–1_01_) transition occurs. The increase in dust optical depth with wavelength leads to an increased availability of 108-μm photons relative to 538-μm photons compared with the thermal equilibrium case of a single-black-body radiation field. This implies that the 2_21_–1_10_ transition at 108 μm is exposed to a more intense infrared radiation field than the 1_10_–1_01_ transition at 538 μm. For the infrared radiation field of HFLS3, the models therefore suggest that both H_2_O transitions should be found in absorption, but that the 2_21_–1_10_ transition (which is not covered by our observations) will contribute a larger fraction to the 1_10_ level de-population than that found for the thermal equilibrium case. The CMB photons, on the other hand, only provide the base population expected for the thermal black-body equilibrium case. This causes a reduced excitation temperature in the 1_10_–1_01_ transition compared with the thermal equilibrium case, unless the 1_01_ level is even more substantially de-populated due to the H_2_O(2_12_–1_01_) transition at 180 μm. This, however, does not occur, as our models show that this transition is expected to be seen in emission (at a line strength approximately 3–5 times below a previously reported upper limit^5^). This is because the upper-level population of the 2_12_–1_01_ transition is also affected by the population of the 2_12_ level through the 2_21_–2_12_ line, which appears in emission due to the pumping of its upper 2_21_ state by the 2_21_–1_10_ absorption line. As such, the models suggest a net deficit in the upper-level population of the 1_10_–1_01_ transition compared with the thermal equilibrium case, which causes the *T*_ex_ of the line to end up below the CMB temperature.

**Extended Data Fig. 2:**
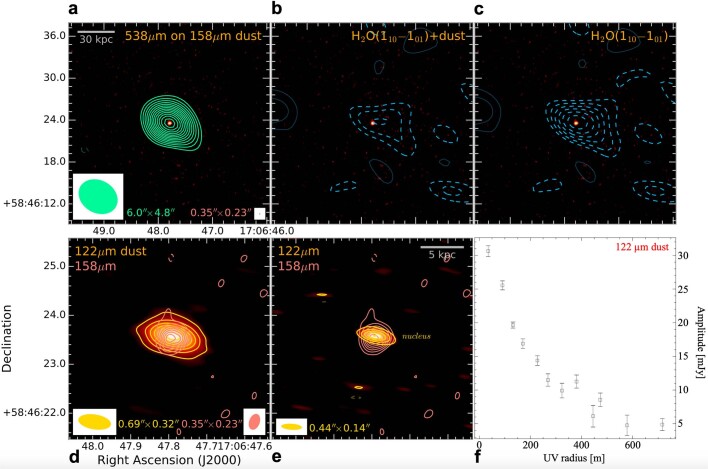
H_2_O line emission integrated moment 0 and continuum maps of HFLS3. **a**–**c**, H_2_O contour maps (blue) before (**b**) and after (**c**) continuum subtraction, and local continuum (**a**, green contours) at the wavelength of the H_2_O line, overlaid on the 158-μm continuum (intensity scale)^5^. H_2_O emission is integrated over the central 395 km s^−1^ (100 MHz). **d**–**e**, Rest-frame 122-μm continuum emission (orange contours and intensity scale) as a proxy for the 108-μm continuum size, showing the full emission (**d**), and the compact nuclear region that accounts for two-thirds of the emission at higher resolution (**e**), overlaid with 158-μm contours (red) for orientation. **f**, Radially averaged visibility amplitude as a function of interferometer baseline length for the data in **d** and **e**. The radial profile of the visibility amplitude (binned to 50-m steps, with 1*σ* error bars) shows that the 122-μm dust emission is clearly resolved. Observed-frame 538-μm continuum contours (**a**) are shown in steps of 1*σ* = 22.5 μJy beam^−1^, starting at ±3*σ*. H_2_O contours (**b**, **c**) are shown in steps of 1*σ* = 0.0375 Jy kms^−1^ beam^−1^, starting at ±2*σ*. Contours of 122 μm (**d**, **e**) are shown in steps of +/−10*σ* and +/−5*σ*, where 1*σ* = 229 and 374 μJy beam^−1^, respectively. Contours of 158 μm (**d**, **e**) are shown in steps of 3*σ*, starting at ±5*σ*, where 1*σ* = 400 μJy beam^−1^ (all uncertainties are r.m.s.). Negative intensity contours are dashed.

The RADEX models yield *T*_ex_ = 17.4 K due to this level-population modification. To translate model-predicted temperature differences into an absorption-line flux that can be compared with the observations, the size of the emitting/absorbing region needs to be known. Based on NOEMA observations at rest-frame 122 μm (Extended Data Fig. 2), we estimate the dust continuum size of the emitting region at 108 μm (the wavelength of the pumping transition) of HFLS3 to be *r*_108μm_ = 1.62 ± 0.45 kiloparsecs (kpc). Within the uncertainties of the size estimate, the RADEX models suggest that the strength of the observed H_2_O absorption can be reproduced over about two orders of magnitude in H_2_O column density, with a lower limit of around 10^16^ cm^−2^. The minimum covering fraction of the dust continuum is about 60% when conservatively leaving *T*_CMB_ as a free parameter (100% is assumed for the grid shown in Fig. 3a). The upper limit for the H_2_ column density implied by the gas mass of HFLS3 of (1.04 ± 0.09) × 10^11^ M_sun_ (ref. ^5^) provides a lower limit to the gas phase [H_2_O]/[H_2_] abundance of >2 × 10^−7^, which falls within the range of 10^−9^–10^−5^ found for nearby starbursts^11^. The small difference Δ*T* = *T*_ex_ − *T*_CMB_ = −2.6 K is, therefore, sufficient to explain the observed strength of the H_2_O(1_10_–1_01_) absorption line towards the CMB in HFLS3 when assuming a layer of cold, diffuse H_2_O-bearing gas with a high covering fraction in front of the warm dust continuum source associated with the H_2_O emission lines.

As the absorption line is observed in contrast to the CMB, we can use the strength of the absorption line to obtain a measurement of *T*_CMB_ at the redshift of HFLS3. The RADEX models suggest that, to detect the H_2_O(1_10_–1_01_) line in absorption against the CMB, *T*_CMB_(*z* = 6.34) must be >7–8 K, independent of the model assumptions (see Methods). The observed strength of the signal suggests 16.4 K < *T*_CMB_(*z* = 6.34) < 30.2 K (1*σ*, or 12.8 K < *T*_CMB_(*z* = 6.34) < 34.0 K 2*σ*) for HFLS3 when treating *T*_dust_, *β*_IR_ and the wavelength where the dust optical depth reaches unity as free-fitting parameters for each dust continuum size sampled by the models. This explains why the effect has not been previously seen. *T*_CMB_ must be sufficiently high to satisfy the requirement of a notable H_2_O 1_10_ level population due to the CMB, such that a de-population by the infrared radiation field of the starburst will lead to a sufficiently important decrement to be observable in absorption against the CMB. This limits observability to *z* > 4.5 for dust spectral energy distribution shapes and dust continuum sizes of star-forming galaxies like HFLS3 (Fig. 3c), where only a few spectra at rest-frame 538 μm with sufficient signal-to-noise ratio to detect the effect exist. This differs from molecules like H_2_CO, for which absorption against the CMB has been predicted to occur at any redshift up to the present day^12^, but for which no detections at high redshift currently exist^13^. For starbursts with dust as warm as HFLS3, its relative strength is expected to continue to increase with redshift all the way up to *z* ~ 7–8, and to remain observable back to the earliest epochs when such galaxies existed.

The thermal Sunyaev–Zel’dovich (SZ) effect entails the modification of the thermal distribution of CMB photons by Thomson scattering off electrons at high temperatures in the intergalactic medium of galaxy (proto)clusters. The effect observed here requires the CMB photons to excite the H_2_O rotational ladder to create a thermal distribution of the lower energy levels, which is then modified through the absorption of far-infrared photons from the starburst radiation field permeating the interstellar medium. In both cases, the CMB is responsible for establishing unperturbed thermal distributions (of photons and H_2_O excitation, respectively), which are then modified by local conditions. The SZ effect is a broad-band modification of the thermal distribution of CMB photons via scattering, with an expected signal strength (relative to the CMB) that is independent of redshift. In contrast, the H_2_O absorption signal described herein is a narrow-band (spectral-line) absorption process of the CMB photons, catalysed (in part) by the CMB itself, with an absorption-line strength that increases with redshift owing to the increasing temperature of the CMB, relative to the fixed excitation temperature of the H_2_O(1_10_–1_01_) transition.

**Fig. 4:**
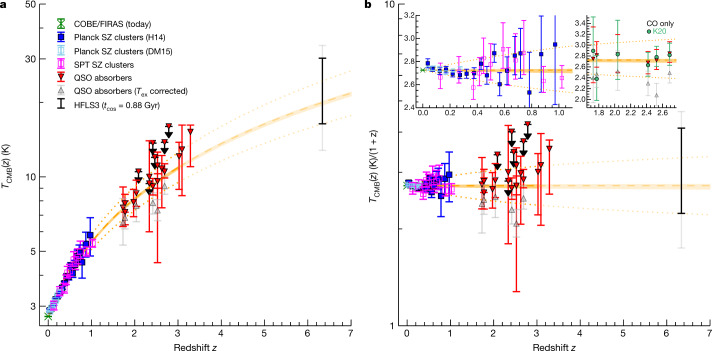
Measurements of the CMB temperature as a function of redshift^3,17,19–28^. **a**, 1*σ* (black) and 2*σ* r.m.s. (grey) uncertainties are shown for HFLS3 and 1*σ* r.m.s. uncertainties elsewhere**. b**, Same data but dividing out the (1 + *z*) redshift scaling of the CMB expected from ΛCDM. Previous direct measurements are from CMB mapping at *z* = 0 and SZ effect measurements of galaxy clusters in contrast to the CMB out to *z* ~ 1 (left zoom-in panel in **b**). Additional measurements are from ultraviolet absorption systems along the lines of sights to quasars out to *z* ~ 3. The downward (upward) triangles are not corrected (corrected) for the contribution of collisional excitation in the diffuse interstellar medium to the excitation temperature *T*_ex_ of the tracer (right zoom-in panel in **b**; green dots show an alternative proposed correction^3^)^21,27^. The separation of these pairs of points for the same sources is an indication of the systematic uncertainties on top of the statistical uncertainties indicated by the error bars. The H_2_O-based measurement of HFLS3, like those up to *z* ~ 1, is in contrast to the CMB, but—as a line measurement—it is more precise in redshift. It is not subject to the same uncertainties in *T*_ex_ as the intermediate redshift measurements, because collisions can only decrease (rather than boost) the resulting absorption strength into the CMB for the H_2_O-based measurement. They are also unlikely to play an important role due to the high density required to collisionally excite the relevant H_2_O lines. Ignoring collisions results in the most conservative estimate of *T*_CMB_ for HFLS3. The orange shaded region shows a *T*_CMB_ = *T*_CMB_(*z* = 0)*(1 + *z*)^1 − *β*^ fit to the data in Extended Data Table 1 and its uncertainty (where *T*_CMB_(*z* = 0) = 2.72548 ± 0.00057 K (ref. ^7^) and *β* = (${3.4}_{-7.3}^{+8.1}$) × 10^−3^), the orange dashed line indicates the *β* = 0 case corresponding to the standard cosmology and the dotted lines indicate a ±10% deviation in 1 − *β*.

Standard ΛCDM cosmologies predict a linear increase of *T*_CMB_ with (1 + *z*). However, there are hypothetical physical mechanisms that could lead to departures from this linear behaviour, including the evolution of physical constants^14^, decaying dark-energy models^15^ and axion-photon-like coupling processes^16,17^. Direct measurements of *T*_CMB_ are, thus, a crucial test of cosmology, but they are currently limited to *z* < 1, due to the lack of sufficiently precise measurements of the thermal SZ effect in galaxy clusters at higher redshifts (Fig. 4; see Methods for further details). A limited sample of additional constraints exists at *z* = 1.8–3.3 based on measurements of *T*_ex_ for the ultraviolet transitions of CO, [CI] and [CII] in absorption-line systems along the lines of sight to quasars. These lines are not directly observed in contrast to the CMB, and they use the *T*_ex_ of these lines as a proxy of *T*_CMB_, such that the resulting *T*_CMB_ estimates are subject to model-dependent excitation corrections^3,17–28^. As an example, for the CO molecule, *T*_ex_ typically already exceeds *T*_CMB_ in the diffuse interstellar medium in the Milky Way due to collisional excitation, showing a rising excess with increasing CO optical depth due to photon trapping^18^. In contrast, our models suggest that collisional excitation of H_2_O becomes important only at very high densities, such that H_2_O-based measurements are probably only minimally affected by this effect. The H_2_O absorption against the CMB at *z* = 6.34 reported here thus provides the most direct constraint on *T*_CMB_ currently available at *z* > 1. Indeed, the existence of this effect on its own directly implies that the CMB is warmer than at low redshift, because *T*_CMB_ must be sufficiently high to notably excite the H_2_O 1_10_ level, which lies 26.7 K above ground, as a basis for the observed decrement due to de-population of this level by the starburst radiation field. A combined fit to the available data (Fig. 4) is consistent with the redshift scaling expected from ΛCDM. Fitting for the adiabatic index *γ* in the equation of state between pressure *P* and energy density *ρ* for the sum of baryonic and dark matter and radiation—that is, *P*_rm_ = (*γ* − 1)*ρ*_rm_ with a standard formalism (see Methods)—we find *γ* = ${1.328}_{-0.007}^{+0.008}$, which agrees with the standard value of *γ* = 4/3 expected in ΛCDM. At the same time, we find an effective dark energy equation of state parameter *w*_eff_ = *P*_de_/*ρ*_de_ = −${1.011}_{-0.017}^{+0.018}$, which is consistent with the *w* = −1 expectation for a dark energy density that does not evolve with time.

## Methods

### NOEMA observations

The target was observed in the 3-mm wavelength band 1 (rest-frame 400 μm) with NOEMA as part of project S20DA (Principal Investigators: D. A. Riechers, F. Walter). Three partially overlapping spectral setups were observed under good weather conditions between 26 July 2020 and 25 August 2020 with ten antennas in the most compact D configuration, using a bandwidth of 7.7 GHz (dual polarization) at 2-MHz spectral resolution per sideband. We also included previously published^5^ observations between 6 February 2012 and 31 May 2012 in the A and D configurations tuned to 110.128 and 113.819 GHz, respectively, and previously unpublished observations between 1 June 2012 and 4 June 2012 and on 10 July 2017 in the D configuration tuned to 78.544 and 101.819 GHz taken as part of projects V0BD, W058, and S17CC (Principal Investigator: D. A. Riechers), all using 3.6 GHz of bandwidth (dual polarization), yielding 21 observing runs in total. Nearby radio quasars were used for complex gain, bandpass and absolute flux calibration. The target was also observed in the 0.87-mm wavelength band 4 (rest-frame 122 μm) with NOEMA as part of project X0CC (Principal Investigator: D. A. Riechers). Observations were carried out during three observing runs with six antennas in the A and C configurations under good weather conditions between 4 December 2013 and 12 March 2015, with the band 4 receivers tuned to 335.5 GHz and using a bandwidth of 3.6 GHz (dual polarization). Nearby radio quasars were used for complex gain, bandpass and absolute flux calibration. The GILDAS package was used for data calibration and imaging. All 3-mm data were combined to a single visibility cube before imaging. Imaging was carried out with natural baseline weighting. The band 4 data were also imaged with Briggs robust weighting to increase the spatial resolution. A map of the continuum emission at the frequency of the H_2_O line was created by averaging the visibility data over a bandwidth of 2.04 GHz centred on the line. This range was chosen to avoid other lines in the bandpass. Continuum emission was subtracted from the H_2_O line cube in the visibility plane. Moment 0 images of the line absorption were created before and after continuum subtraction by integrating the signal over a bandwidth of 100 MHz, corresponding to 395 km s^−1^. The resulting r.m.s. noise levels are provided in Extended Data Fig. 2. We also make use of previously published^5^ rest-frame 158-μm NOEMA data, which were adopted without further modification.

### Line and continuum parameters

The flux of the H_2_O(1_10_–1_01_) line was extracted by simultaneous Gaussian fitting of the line and continuum emission (including a linear term for the continuum) in the one-dimensional spectrum shown in Fig. 1, which was extracted from the image cube. The source is unresolved at the frequency of the H_2_O(1_10_–1_01_) line, such that the main uncertainties are due to the slope of the continuum emission and the appropriate fitting of other nearby lines, in particular, CO(5–4). The uncertainties in these parameters are part of the quoted uncertainties. We find a line peak flux of −818 ± 145 μJy at a line full width half maximum (FWHM) of 507 ± 111 km s^−1^, centred at a frequency of 75.8948 GHz (±46 km s^−1^; the calibration uncertainties on the line FWHM and centre frequency are negligible and that on the line peak flux is <10%—that is, minor compared with the measurement uncertainty). Given the rest frequency of the line of 556.9359877 GHz, this corresponds to a redshift of 6.3383, which is consistent with the systemic redshift of HFLS3 (*z* = 6.3335 and 6.3427 with uncertainties of ±14 and ±54 km s^−1^ at Gaussian FWHM of 243 ± 39 and 760 ± 152 km s^−1^, respectively, for the two velocity components detected in the 158-μm [CII] line)^5^. For comparison, the H_2_O(2_02_–1_11_) and H_2_O(2_11_–2_02_) emission lines in HFLS3 have FWHM of 805 ± 129 and 927 ± 330 km s^−1^, respectively^5^—that is, only marginally broader than the 1_10_–1_01_ line at the current measurement uncertainties. The continuum flux at the line frequency is 396 ± 15 μJy, corresponding to 48% ± 9% of the absorption-line flux (the relative flux calibration uncertainty between the line and continuum emission is negligible). We also measured the 335.5-GHz continuum flux by two-dimensional fitting to the continuum emission in the visibility plane. We find a flux of 33.9 ± 1.1 mJy, which agrees with previous lower-resolution observations at the same wavelength^5^. The major (minor) axis FWHM diameter of the source is 0.617 ± 0.074 arcsec (0.37 ± 0.20 arcsec). This yields the physical source size quoted in the main text at the redshift of HFLS3.

### Brightness temperature contrast

The H_2_O(1_10_–1_01_) line leads to a decrement in continuum photons from the starburst and, as such, is observed as a lack of continuum emission at its frequency at the position of the starburst. It therefore appears as negative flux in an image where starburst continuum emission has been subtracted. In addition, (sub)millimetre-wavelength interferometric images reveal structure against a flat sky background defined by the large-scale CMB surface brightness, which the interferometer does not detect itself due to its limited spatial sampling. Therefore the fraction of the signal due to the decrement in CMB photons at the position of the starburst not only appears as negative flux without subtracting any further signal but it also corresponds to a lack of continuum emission at the line frequency in practice. As the mere presence of an absorption-line signal stronger than the measured continuum emission implies absorption against the CMB, this interpretation is not limited by uncertainties in the galaxy continuum flux or uncertainties in the absolute flux calibration.

### Line-excitation modelling

RADEX is a radiative transfer program to analyse interstellar line spectra by calculating the intensities of atomic and molecular lines, assuming statistical equilibrium and considering collisional and radiative processes, as well as radiation from background sources. Optical depth effects are treated with an escape probability method^8^. Studies of nearby star-forming galaxies show that the observed absorption strengths of the ground-state H_2_O and H_2_O^+^ transitions are due to cooler gas that is located in front of, and irradiated by, a warmer background source that is emitting the infrared continuum light that also excites the higher-level H_2_O emission lines^11,29^. We therefore adopt the same geometry for the modelling in this work, which is adequately treated within RADEX (that is, treating the dust continuum plus the CMB as background fields for the absorbing material)^8^. The dust continuum emission is modelled as a grey body with treating *T*_dust_, *β*_IR_ and the wavelength where the dust optical depth reaches unity as free-fitting parameters for each dust continuum size and *T*_CMB_ sampled by the models. The observed spectral energy distribution of HFLS3, including all literature^5^ photometry and the measurements presented in this work, is then treated as the contrast between the dust continuum and CMB background fields, such that the resulting fit parameters for the dust continuum source change with *T*_CMB_ in a self-consistent manner. In the RADEX models, we derive the H_2_O peak absorption depth into the CMB. We then multiply the best matching peak absorption depth found by RADEX with a Gaussian matched to the fitted line centroid and line width obtained from the observed line profile in Fig. 2 to determine the model line profile. In this approach, the shallower absorption in the line wings either corresponds to a lower filling factor of the H_2_O layer at the corresponding velocities or to lower H_2_O column densities. Although collisions of H_2_O molecules with H_2_ is another mechanism that can modify the level populations especially at very high gas densities (which is an important mechanism for the cooling of low-excitation-temperature transitions of molecules like H_2_CO to below *T*_CMB_)^12,30^, the RADEX models show that they do not affect our findings (see Fig. 3c). We therefore adopt models with essentially no collisions by assuming a very low gas density of *n*(H_2_) = 10 cm^−3^. We then compare our findings to those obtained when adopting conditions that are similar to those found in local starburst galaxies^11^ and to those found for high-density environments with *n*(H_2_) > 10^5^ cm^−3^. The cross sections for collisions out of the 1_01_ level are always larger than those out of the 1_10_ level, independent of the collision partner and the temperature at which the collisions take place^31–33^. Therefore collisions cannot be responsible for an over-proportional de-population of the 1_10_ level relative to the 1_01_ ground state, and the net effect of including collisions is a decrease in the absorption depth into the CMB by reducing the *T*_CMB_ − *T*_ex_ temperature difference at very high gas densities compared with cases without collisions. For reference, the effect of collisions on the determination of *T*_CMB_ is negligible for the typical conditions found in local starbursts (that is, *n*(H_2_) ~ 10^4^ cm^−3^; *T*_kin_ = 20–180 K)^11^ and only starts to have an impact for very high densities *n*(H_2_) > 10^5^ cm^−3^. For a given continuum source size, the constraints on *T*_CMB_ would therefore be tighter (that is, would more quickly become inconsistent with the observations) for the high-density case than for the case without collisions, such that the latter approach is more conservative (see Fig. 3b). The overall impact of collisional excitation would therefore be more stringent requirements on the source size, covering fraction and water column, such that their inclusion would only further strengthen our conclusions. We note that this is the opposite effect to the case of the studies of ultraviolet lines^3,17,19–28^, where neglecting collisional excitation results in less conservative constraints on *T*_CMB_. If we were to assume that the H_2_O absorption were to emerge from within the infrared continuum-emitting region, a larger source size would probably be required to obtain the same absorption-line strength due to a reduced effective radiation field strength from the starburst. Previous modelling attempts of nearby galaxies assuming such geometries have not been able to produce H_2_O(1_10_–1_01_) line absorption on the scales necessary to explain the observations of HFLS3, which may indicate that even more complex assumptions would be required^11^. Thus, the resulting constraints would, once again, be less conservative, perhaps acting in a similar manner as the high-density case. Excluding both of these effects from the models leads to a maximally conservative estimate of *T*_CMB_ and its uncertainties. Assuming a plane-parallel or similar geometry instead of a spherical geometry would only have a minor impact on our findings^8^. The models shown in Fig. 3 assume a filling factor of unity, which is the most conservative possible assumption. A more clumpy geometry with a lower covering fraction remains possible for all *T*_CMB_ values for which the predicted absorption strength exceeds the observed value (see shaded regions in Fig. 3b). For reference, the minimum covering fractions consistent with the continuum size at the observed signal strength are shown for the different cases considered in Fig. 3. The line absorption is also found to be optically thick, with an optical depth of *τ*_H2O_ = 21.1 for the solution shown in Fig. 2b. To determine the redshift above which the effect becomes observable (Fig. 3c), we fixed *r*_108μm_, *T*_dust_, *β*_IR_ and *M*_dust_ to the observed values and the H_2_O column density to the value corresponding to the model spectrum. H_2_O line absorption into the dust continuum of HFLS3 would already become visible at *z* > 2.9, but absorption into the CMB only becomes observable at *z* > 4.5 (or higher for H_2_ densities of >10^5^ cm^−3^). These values account for changes in the shape of the dust grey-body spectrum (that is, changes in the relative availability of 538-μm and 108-μm photons) due to changes in *T*_CMB_ with redshift. To better quantify the impact of different modelling parameters, we have varied *T*_dust_ and *β*_IR_ beyond their previously estimated uncertainties (nominal reference values without considering variations in *T*_CMB_ from the literature are *T*_dust_ = ${63.3}_{-5.8}^{+5.4}$ K and *β*_IR_ = ${1.94}_{-0.09}^{+0.07}$)^5,6^. This is necessary because both parameters are dependent on the varying *T*_CMB_ in our models (and therefore are changing parameters in Fig. 3b, c), such that their true uncertainties need to be re-evaluated. We independently varied *β*_IR_ in the 1.6–2.4 range and *T*_dust_ in the ±20 K range as functions of *T*_CMB_ around the best-fit values. This shows that *β*_IR_ > 2.0 and *T*_dust_ lower by more than 10 K from the best fits yield very poor fits to the spectral energy distribution data, whereas Δ*β*_IR_ > −0.1 below the best-fit value would require a larger continuum size than the measured *r*_108μm_ + 1*σ* and therefore are disfavoured by the size constraint. Excluding these ranges, the extrema across this entire range would extend the uncertainty range in the predicted *T*_CMB_ by only −1.7 and +5.4 K and −0.8 and +4.4 K for the *r*_108μm_ + 1*σ* and *r*_108μm_ + 2*σ* cases, respectively. For comparison, the difference between the +1*σ* and +2*σ* uncertainty ranges is −3.6 and +3.8 K). This shows that the impact of the uncertainties in the dust spectral energy distribution fitting parameters on those in *T*_CMB_ are subdominant to those in the continuum size measurement. Conversely, we have studied the impact of changes in *T*_CMB_ on the best-fit *T*_dust_ and *β*_IR_. For the values corresponding to *r*_108μm_ + 1*σ* and *r*_108μm_ + 2*σ* ranges, *T*_dust_ typically changes by <0.5 K and *β*_IR_ typically changes by <0.1–0.2 when varying the parameters independently. These changes are larger than the actual uncertainties, because the fit to the dust spectral energy distribution becomes increasingly poorer with these single-parameter variations. At the same time, these changes are subdominant to those induced by changes in dust continuum size within the +1*σ* and +2*σ* uncertainty ranges, which is consistent with our other findings.

### Other H_2_O transitions in HFLS3

Five H_2_O lines were previously detected towards HFLS3 (2_02_–1_11_, 2_11_–2_02_, 3_12_–2_21_, 3_12_–3_03_ and 3_21_–3_12_) and two additional lines were tentatively detected (4_13_–4_04_ and 4_22_–4_13_)^5^. The *J*_up_ = 3 transitions are due to ortho-H_2_O and all other transitions are due to para-H_2_O. All of these transitions appear in emission. Given the high critical densities of these transitions, our RADEX models cannot reproduce the strength of these lines as the same time as the observed ortho-H_2_O(1_10_–1_01_) absorption strength, which suggests that they emerge from different gas components. For reference, to reproduce the strength of the H_2_O(2_11_–2_02_) in Fig. 1 alone with collisional excitation, *n*(H_2_) = 2 × 10^7^ cm^−3^ and *T*_kin_ = 200 K would be required, but the H_2_O(1_10_–1_01_) would no longer appear in absorption against the CMB if it were to emerge from the same gas component. This is consistent with the picture that the H_2_O absorption is due to a cold gas component along the line of sight to the warm gas that gives rise to the emission lines^11^. Observations of the para-H_2_O(1_11_–0_00_) ground state do not currently exist for HFLS3, but our models do not show this line in absorption towards the CMB.

### Origin of the lower and upper limits on *T*_CMB_

Our models show that the lower limit on *T*_CMB_ at a given redshift based on the observed H_2_O absorption is due to the minimum ‘seed’ level population due to the CMB black-body radiation field. To determine a conservative lower limit, we have calculated models with continuum sizes up to *r*_108μm_ = 5 kpc (see Fig. 3b), corresponding to a +7.5*σ* deviation from the observed continuum size, and recorded the temperatures at which such weakly constrained models turn into absorption. We find that this results in a lower limit of *T*_CMB_ > 7–8 K, independent of the model assumptions. This finding alone does not explain the existence of an upper limit in Fig. 3b. For a given size of the dust continuum emission, an increase in *T*_CMB_ also requires an increase in *M*_dust_ to still reproduce the observed dust spectral energy distribution, which leads to an effective increase in the dust optical depth at a given wavelength. The result of a rising optical depth is that the grey-body spectrum between 538 and 108 μm increasingly resembles a black-body spectrum and, hence, a decrease in the H_2_O absorption against the CMB. This effect is responsible for the upper limit in allowed *T*_CMB_ for a given dust continuum size and absorption strength.

### Uncertainties of *T*_CMB_ measurements

The uncertainties shown for the literature data in Fig. 4 are adopted from the literature sources without modification, and they typically represent the statistical uncertainties from the individual measurements or sample averages. Individual cluster measurements of the thermal SZ effect may be affected by dust associated with foreground galaxies or the Milky Way, the galaxy clusters or background galaxies that may be amplified by gravitational lensing, uncertainties in the reconstruction of the Compton-*y* parameter maps due to flux uncertainties, radio emission due to active galactic nuclei and/or relics, the kinetic and relativistic SZ effects, and general bandpass and calibration uncertainties^17^. Furthermore, uncertainties on the cluster geometry—and therefore line-of-sight travel distance of the CMB photons through the cluster—and on the temperature of the intra-cluster gas limit the precision of individual SZ measurements. Sample averages may also be affected by systematics in the stacking procedures. Individual data points deviate by up to at least two standard deviations from the trend, which may indicate residual uncertainties beyond the statistical error bars provided, such that the error bars shown in Fig. 4 are underestimated. The main source of uncertainty for the ultraviolet absorption-line-based measurements are due to the assumption of no collisional excitation, which is not taken into account in the statistical uncertainties shown in Fig. 4. Attempts to take this effect into account appear to suggest substantially larger uncertainties than indicated by individual error bars^27^ (Fig. 4). To expand on earlier estimates^21^, we have calculated RADEX models for typical *T*_kin_, *n*(H) and column densities found from [CI] measurements in the diffuse interstellar medium^34^, which suggests that collisional excitation contributes to the predicted *T*_ex_ of the lower fine-structure transition. Although we show the original unmodified data, the ultraviolet-based measurements are therefore subject to uncertainties due to model-dependent excitation corrections in addition to the statistical uncertainties. Furthermore, the fine-structure levels of tracers like the [CI] lines can be excited by ultraviolet excitation and following cascades. To constrain *T*_CMB_ based on these measurements, the kinetic temperature, particle density and local ultraviolet radiation field must be known, and are typically determined based on tracers other than the species used to constrain *T*_CMB_. Also, some measurements are based on spectrally unresolved lines, which limits the precision of kinetic temperature measurements based on thermal broadening^21^. Owing to these uncertainties, the ultraviolet absorption-line-based measurements are probably consistent with the standard ΛCDM value, but they do not constitute a direct measurement of *T*_CMB_ without notable further assumptions. For reference, the median *T*_CMB_/(1 + *z*) estimate based on the [CI] measurements alone (excluding upper limits) is 3.07 K, with a median absolute deviation of 0.09 K and a standard deviation of 0.31 K. Therefore the current sample median deviates from the ΛCDM value by about one standard deviation. A combination of the (uncorrected) measurements based on CO, [CI] and [CII] provides a median value of 2.84 K, with a median absolute deviation of 0.15 K and a standard deviation of 0.25 K. This highlights the importance of the corrections discussed above and in the literature and the value of measurements with systematic uncertainties that differ from this method to obtain a more complete picture. The main source of uncertainty of the H_2_O-based measurements, beyond the caveats stated in the line-excitation-modelling section, are the statistical uncertainties on the source size, the lack of a direct measurement of the absorbing H_2_O column density, variations in the dust mass absorption coefficient and the filling factor. Given the high metallicity suggested by other molecular line detections, the limitation to high filling factors due to the source size and the constraint on the gas mass from dynamical mass measurements, the main source of uncertainty resides in the source size due to limited spatial resolution in the current data. As such, major improvements should be possible by obtaining higher, (sub-)kpc resolution (that is, <0.2”) imaging with the Atacama Large Millimeter/submillimeter Array (ALMA; for other targets) and planned upgrades to NOEMA, and, in the future, with the Next Generation Very Large Array (ngVLA). Statistical uncertainties will also be greatly reduced by observing larger samples of massive star-forming galaxies over the entire redshift range where measurements are possible, closing the gap to SZ-based studies, which are currently limited to *z* < 1. The resulting improvement in precision will provide the constraints that are necessary to confirm or challenge the evolution of the CMB temperature with redshift predicted by standard cosmological models.

### Accessibility of the line signal

The frequency range currently covered by NOEMA is 70.4–119.3, 127.0–182.9 and 196.1–276.0 GHz (with greatly reduced sensitivity above about 115 and 180 GHz in the first two frequency ranges). ALMA covers the 84–500-GHz range with gaps at 116–125 and 373–385 GHz, with a future extension down to 65 GHz (with greatly reduced sensitivity below about 67 GHz). The ngVLA is envisioned to cover the 70–116-GHz range. Excluding regions of poor atmospheric transparency, the H_2_O(1_10_–1_01_) line is therefore observable in these frequency ranges at redshifts of *z* = 0.1–0.4, 0.5–2.0, 2.1–3.4 and 3.8–6.9 in principle, but the detectability of the line in absorption against the CMB is probably limited to the *z* ~ 4.5–6.9 range if the spectral energy distribution shape of HFLS3 is representative. At lower frequencies, the Karl G. Jansky Very Large Array and, in the future, ALMA and the ngVLA also provide access to the <52-GHz range, such that the signal also becomes observable at *z* > 9.7 in principle. In conclusion, the absorption of the ground-state H_2_O transition against the CMB identified here could be traced from the ground towards star-forming galaxies across most of the first approximately 1.5 billion years of cosmic history.

### Detectability of the line signal for different spectral energy distribution shapes

To investigate whether the effect is expected to be detectable for different galaxy populations, we have applied our modelling to the *z* = 3.9 quasar APM 08279+5255, for which the dust spectral energy distribution is composed of a dominant 220-K dust component and a weaker 65-K dust component, contributing only 10–15% to the far-infrared luminosity^35–46^. The models suggest that the line is expected to occur in emission and that it would not be expected to be detectable in absorption at any redshift out to at least *z* = 12 in galaxies with similar dust spectral energy distributions. Other far-infrared-luminous, high-redshift, active galactic nucleus host galaxies typically show a stronger relative contribution of their lower-temperature dust components, such that the effect may remain detectable in less extreme cases. For galaxies with lower dust temperatures than HFLS3, the effect may be present even at lower redshifts, but is typically expected to be weaker in general and to disappear at redshifts where *T*_CMB_ approaches their *T*_dust_. For a dust spectral energy distribution shape resembling the central region of the Milky Way but otherwise similar properties, the effect is expected to be reduced by more than two orders of magnitude at its redshift peak, and to become virtually unobservable at the redshift of HFLS3. Thus, dusty starburst galaxies appear to be some of the best environments to detect the effect.

### Derivation of equation of state parameters

To determine the adiabatic index, we assume a standard Friedmann–Lemaitre–Robertson–Walker cosmology with zero curvature and a matter-radiation fluid that follows the standard adiabatic equation of state quoted in the main text. This would correspond to a redshift scaling *T*_CMB_(*z*) = *T*_CMB_(*z* = 0)*(1 + *z*)^3(*γ* − 1)^ in the presence of a dark energy density that does not scale with redshift. The dark energy density is parameterized to scale with a power law (1 + *z*)^*m*^, where *m* = 0 corresponds to a cosmological constant. With standard assumptions, this yields a redshift scaling of *T*_CMB_ (ref. ^15^):

$${T}_{CMB}(z)={T}_{CMB}(z=0){(1+z)}^{3(\gamma -1)}{\left[\frac{(m-3{\varOmega }_{m,0})+m{(1+z)}^{(m-3)}({\varOmega }_{m,0}-1)}{(m-3){\varOmega }_{m,0}}\right]}^{(\gamma -1)}$$

and an effective dark energy equation of state *P*_de_ = *w*_eff_*ρ*_de_, where the effective equation of state parameter *w*_eff_ = (*m*/3) − 1. This fitting function is used here with a canonical value of *Ω*_m,0_ = 0.315 (ref. ^4^). The uncertainty of *Ω*_m,0_ is small compared with all other sources of uncertainty and, hence, is neglected. All data used in the fitting are provided in Extended Data Table 1 (refs. ^36–46^).

## Online content

Any methods, additional references, Nature Research reporting summaries, source data, extended data, supplementary information, acknowledgements, peer review information; details of author contributions and competing interests; and statements of data and code availability are available at 10.1038/s41586-021-04294-5.

### Source data

Source Data Fig. 2

## Figures and Tables

**Extended Data Table 1:**
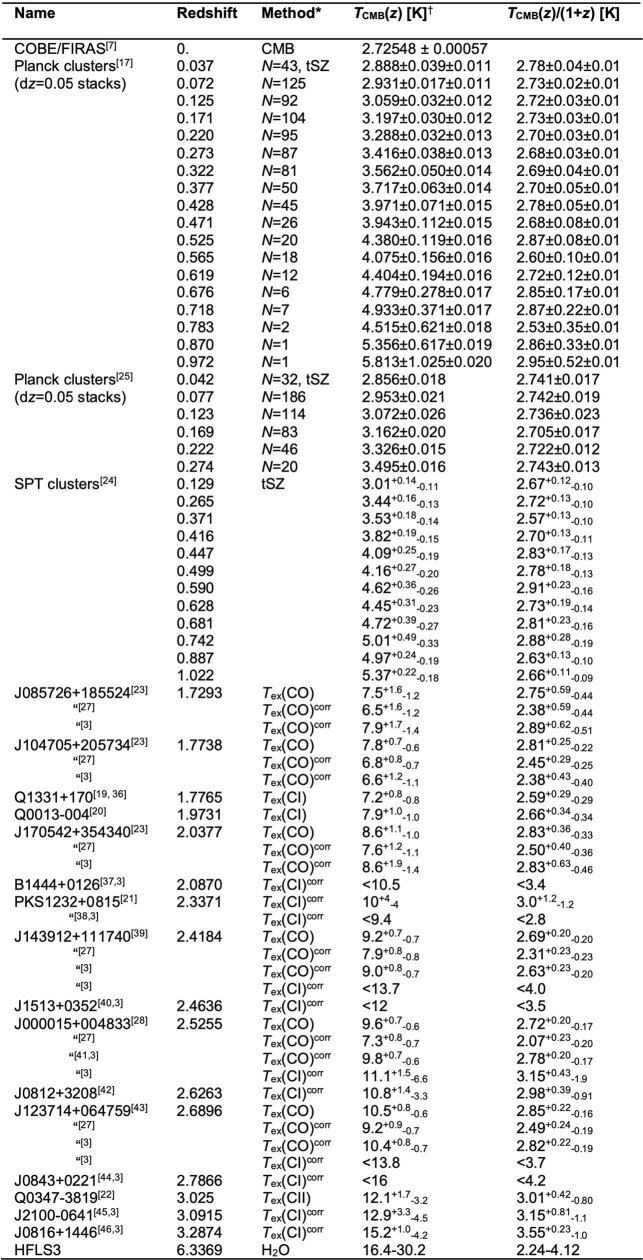
Summary of *T*_CMB_(*z*) measurements

*CMB, measured from the cosmic microwave background; H_2_O, water-based method introduced here; *N*, number of clusters contributing to the stacked measurements; *T*_ex_, indirect measurement based on the excitation temperature of CO, [CI] or [CII] of ultraviolet absorption lines along the lines of sight to background quasars, with model-based excitation corrections applied where indicated (different values indicate model corrections from different authors to the same data); tSZ, measured from the thermal Sunyaev–Zel’dovich effect in galaxy clusters.

^†^Where provided, the second set of error bars indicates systematic uncertainties. Other error bars correspond to statistical uncertainties.

## Data Availability

The spectral line data and model generated and analysed during this study as shown in Fig. 2 are linked to this manuscript in spreadsheet form. Additional versions of the NOEMA datasets (visibilities, images and spectra) are available from the corresponding author (D.A.R.) on reasonable request. All data are also available in the IRAM Science Data Archive (isda@iram.fr) under project IDs V0BD, W058, X0CC, S17CC, and S20DA. Source data are provided with this paper.

## Code availability

The RADEX code used for the modelling presented in this work and shown in Fig. 3 is available at https://home.strw.leidenuniv.nl/~moldata/radex.html.
